# Kinematic determinants of take-off performance in elite and sub-elite male long jumpers: a competition-based two-dimensional motion analysis

**DOI:** 10.3389/fphys.2026.1826666

**Published:** 2026-05-21

**Authors:** Pengfei Wei, Tinggang Yuan, Zhiyu Xie

**Affiliations:** 1 China Institute of Sport Science, Beijing, China; 2 Peking University, Beijing, China

**Keywords:** approach velocity, competition biomechanics, long jump, neuromuscular performance, stretch–shortening cycle, take-off mechanics

## Abstract

**Background:**

The performance of the long jump depends on the effective conversion of high approach velocity into ballistic flight during brief take-off contact. This process requires rapid force production, efficient stretch–shortening cycle (SSC) function, and precise neuromuscular coordination. The approach speed is widely recognized as key determinant of jump distance. The specific kinematic characteristics which distinguish closely matched high-level athletes under real competition conditions remain unclear. Thus, this study was intended for the identification of key kinematic characteristics associated with take-off performance in elite and sub-elite male long jumpers using competition-based two-dimensional motion analysis.

**Methods:**

Twenty-four male long jumpers who performed valid jumps in national-level outdoor competitions were analyzed. The athletes were divided into elite (8.00–8.20 m; n=11) and sub-elite (7.80–7.99m; n=13) groups. High-speed video recordings (100 Hz) was used for quantification of late-approach spatiotemporal parameters of penultimate and last strides, center-of-mass (COM) velocity components at touchdown and take-off. In addition, velocity utilization ratio (ΣTO/ΣTD), and sagittal-plane hip, knee, and ankle joint angles at touchdown (TD), maximum braking (MB), and take-off (TO) were also recorded. Between-group differences were examined using independent-samples tests with Cohen’s d effect sizes.

**Results:**

Elite jumpers demonstrated significantly greater horizontal velocity at take-off than sub-elite athletes (8.63 ± 0.68 vs. 7.77 ± 0.91 m·s^-^¹; *p* = 0.018; *d* = 1.05). Resultant take-off velocity and velocity utilization ratio showed moderate-to-large effect sizes but were not statistically significant (*p* = 0.055–0.057). No significant differences were observed in stride parameters or sagittal-plane joint angles.

**Conclusion:**

Elite long jump performance appears primarily associated with the ability to preserve higher horizontal velocity during take-off, rather than differences in take-off technique. These findings highlight the importance of velocity maintenance and stretch–shortening cycle efficiency during the take-off phase.

## 1 Introduction

The long jump represents a model of high-speed stretch–shortening cycle (SSC) activity in human locomotion. It involves the need to incorporate maximum sprint velocity, rapid production of force, and precise neuromuscular coordination within a single ground contact (Xu et al., 2025a). The horizontal distance covered by the center of mass (COM) determines the performance in the long jump. It is determined by the magnitude and direction of the resultant velocity at the take-off (Wase Mola et al., 2025). The take-off phase is a brief but critical physiological process. In it, the eccentric and concentric activities of large muscles must be synchronized to transform the approach momentum into a ballistic flight. The efficiency of this process is strongly influenced by the mechanical properties of the tendon, which play a key role in energy storage and return during stretch–shortening cycle actions. Thus, understanding the kinematic expression of these neuromechanical processes during competition is essential for bridging laboratory-based insights with real-world performance (Tang et al., 2024; Mangi et al., 2025). In addition, rapid force production during take-off relies on finely tuned neural control mechanisms that regulate the timing and coordination of muscle activation (Wang et al., 2025).

The primary contributory factor of long jump performance is the approach velocity. The greater speed of approach is positively associated with the distance of the long jump. However, the maximization of approach velocity alone does not guarantee superior performance (Yang et al., 2023). This is consistent with established biomechanical models of long jump performance, which identify horizontal velocity at take-off as a primary determinant of jump distance (Hay, 1993; Linthorne, 2008; Graham-Smith and Lees, 2005). The take-off stage involves the athletes providing sufficient vertical force, as well as reduce the horizontal velocity loss due to braking. This is done to achieve a flight trajectory compatible with the optimal projection angle. Such horizontal-vertical velocity trade-off is expressed as the ability of the athlete to sustain a high eccentric force, quickly build the force, and use the elastic energy of the lower limbs musculotendinous system. Consequently, the take-off performance can theoretically be discussed as an indicator of the efficiency of the SSC in the conditions of extreme sprints, assuming (Jayathunga and Chandana, 2022; Baker et al., 2025). From a broader biomechanical perspective, similar principles of energy storage, transfer, and release have also been observed in legged robotic systems, where effective jumping performance depends on coordinated control of compliant structures (Xie et al., 2026).

The final two strides of the approach involve the penultimate and last steps. They play a decisive preparatory role in determining take-off mechanics. The penultimate step is typically characterized by a subtle decrease of COM. It facilitates a more favorable vertical displacement pattern prior to touchdown. The last step then positions the take-off foot to improve the geometry of contact and the stiffness of the joint at impact on the board. These late-approach spatiotemporal adjustments affect the vertical velocity at touchdown and the mechanical demands placed on the hip, knee, and ankle extensors during support. By physiological aspect, they may reflect the preventive modulation of limb stiffness and muscle pre-activation. It enables more effective storage and release of elastic energy. However, evidence remains unclear regarding which stride parameters meaningfully distinguish athletes of similar high-level performance (García-Fresneda et al., 2022; Phukon and Dhar, 2025).

During ground contact, sagittal-plane joint kinematics provide insight into the neuromechanical strategies behind force transmission (Küpper et al., 2023). At touchdown, relatively extended hip and knee angles limit the excessive braking and preserve horizontal momentum. The controlled flexion during early support allows the eccentric energy absorption and storage, especially within the ankle plantar flexors and knee extensors (Harper, 2024). Then, the subsequent rapid extension contributes to the generation of vertical impulse and determines the vertical component of take-off velocity. The joint-angle magnitudes alone do not directly quantify muscle force or power. They serve as observable markers of mechanical strategy, which is employed to negotiate the trade-off between velocity retention & vertical redirection (Yang et al., 2023).

An important and emerging concept which is relevant to exercise physiology is the efficient utilization of velocity during take-off. Instead of examination of horizontal and vertical velocities in isolation, a resultant velocity utilization ratio provide an integrative indicator. It indicates how effectively athletes preserve and redirect mechanical energy during the SSC-dominated support phase. Athletes who are capable of maintaining a higher proportion of their pre-contact velocity have superior eccentric strength, tendon stiffness, and neuromuscular coordination (Pyanzin and Pyanzina, 2020).

Despite considerable substantial biomechanical research in the long jump, there are several research gaps that still exist. First, many studies have examined either world-class finalists or heterogeneous samples that range from wide performance ranges. This makes it difficult for identification of subtle kinematic features that distinguish between closely matched high-level athletes. Secondly, a considerable proportion of current data is derived from laboratory simulations or training environments, It does not replicate the psychological, tactical, and regulatory constraints of official competition. Thus, competition-based analyses are essential to capture valid movement patterns. The advances in high-speed digital video and 2-D motion analysis allows reliable field-based assessment of COM kinematics and joint angles without interference with athlete preparation. From an exercise physiology perspective, the long jump take-off represents an extreme manifestation of fast stretch–shortening cycle function requiring rapid neuromuscular activation, eccentric–concentric muscle interaction, and efficient transmission of force through the muscle–tendon unit. Therefore, analysis of take-off kinematics can provide indirect insight into the physiological mechanisms that underpin explosive human performance during high-intensity locomotion.

While the importance of horizontal velocity in long jump performance is well established, less is known about how this velocity is preserved and expressed during the take-off phase among closely matched high-level athletes under real competition conditions. The present study extends existing literature by examining a homogeneous cohort within a narrow performance range, allowing for the identification of subtle kinematic characteristics that may differentiate athletes beyond gross performance differences. In addition, the inclusion of the velocity utilization ratio provides an integrative perspective on stretch–shortening cycle efficiency during take-off.

Therefore, the purpose of this study was to identify key kinematic determinants of take-off performance in elite and sub-elite male long jumpers using competition-based two-dimensional motion analysis. Specifically, late-approach spatiotemporal characteristics, take-off velocity components, velocity utilization ratio, and sagittal-plane joint kinematics were compared between groups. It was hypothesized that elite athletes would exhibit greater horizontal take-off velocity and more effective preservation of approach speed during the take-off phase.

## 2 Materials and methods

### 2.1 Study design

This study employed a cross-sectional, observational design based on competition-derived video analysis. Take-off kinematics and late-approach spatiotemporal parameters were compared between elite and sub-elite male long jumpers competing within a narrow high-performance range (7.80–8.20 m). No experimental intervention was applied, and all data were collected under official competition conditions.

### 2.2 Participants

The analysis of total of 24 valid competition jumps performed by 24 male long jumpers was performed. The data was collected during official national-level outdoor competitions which were held between 2023 and 2024, including the National Athletics Championships and National Grand Prix series. The participant characteristics of elite and sub-elite (7.80–7.99 male long jumpers are presented in **Table 1**. The video recordings were conducted on-site by the research team by using standardized acquisition procedures in the competitions. For the avoidance of pseudo-replication, one representative jump per athlete was included in the analysis. Specifically, the best valid jump recorded during the competition was selected for each athlete to represent individual performance. The performances equal to or greater than 7.80 m were considered. It guaranteed consistently high technical standard across sample.

**Table 1:** Participant characteristics of elite (8.00–8.20 m) and sub-elite (7.80–7.99 m) male long jumpers.

Variable	Elite (n = 11)	Sub-elite (n = 13)
Age (years)	25.09 ± 4.11	23.92 ± 4.27
Height (cm)	180.27 ± 3.74	183.17 ± 4.39
Body mass (kg)	69.64 ± 6.62	69.58 ± 8.31
Personal Best (m)	8.219 ± 0.117	8.0458 ± 0.198
Seasonal Best (m)	8.079 ± 0.211	7.8755 ± 0.177

Values are presented as mean ± standard deviation (M ± SD).

Based on official competition results, athletes were classified into two performance levels. These two groups were elite group (8.00–8.20 m, n = 11) and sub-elite group (7.80–7.99 m, n = 13). All performances were recorded under official competition conditions, and no experimental intervention was applied. This study utilized and employed non-invasive observational design. This study was reviewed and approved by the Ethics Review Committee of the China Institute of Sport Science, General Administration of Sport of China (ID: 20260206). The procedure was conducted in accordance with institutional guidelines and it conformed to principles of Declaration of Helsinki. Data was collected during official competitions without interference with athlete preparation or performance, and no personal information was reported.

### 2.3 Data collection and video recording

Two dimensional (2D) video-based motion analysis method was employed in capturing and quantification of kinematic characteristics of take-off phase. All video data were recorded by the research team. The recording protocol was standardized and applied on-site under conditions of a real competition. This was done to ensure ecological validity and methodological consistency is maintained. The three high-speed digital cameras (Sony FDR-AX700; 1080p resolution; 100 Hz sampling rate; shutter speed 1/1000 s) made up the recording system. Moreover, it also included adjustable tripods (height range: 1.00-1.50 m), 2-D calibration frame (1.20 m x 1.20 m), laser rangefinder, a levelling tool, and calibration auxiliaries.

**Figure 1:**
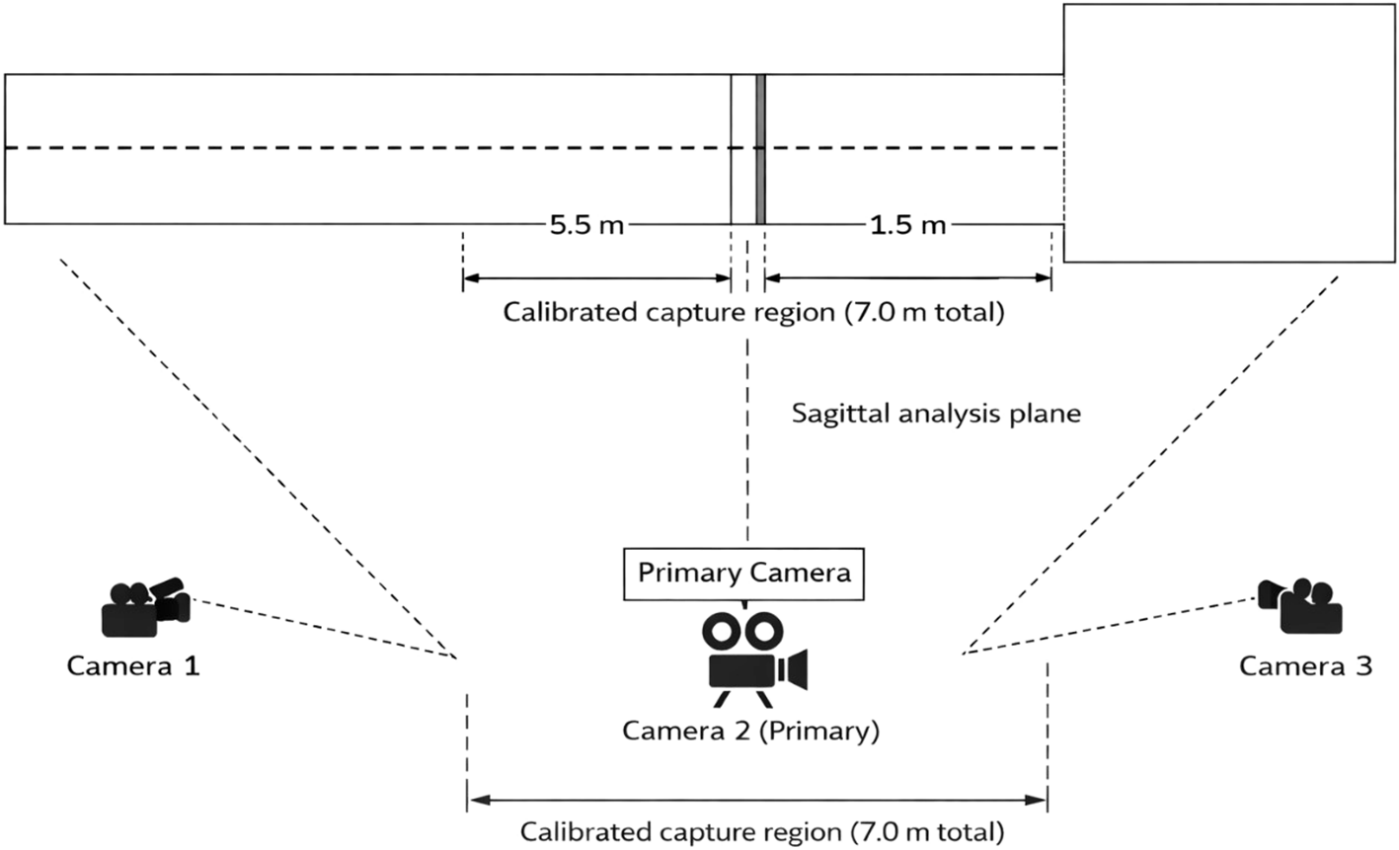
Schematic plan-view layout of the three-camera recording system used during competition. Camera 2 (Primary) was positioned perpendicular to the runway and defined the sagittal analysis plane for two-dimensional kinematic reconstruction. Cameras 1 and 3 were placed obliquely relative to the take-off board and were used solely for event verification and landmark visibility. The calibrated capture region extended 5.5 m before and 1.5 m beyond the take-off board (7.0 m total).

A constant recording design was followed in order to have consistency in the trials (**Figure 1**). There was one main camera which was located about 25m at right angles to the runway. Its optical axis was orthogonal to the direction of approach. This was the only source of data that was used in the reconstruction of kinematics using this camera. It was also a source of field of view that stretched to about 5.5 m, pre take-off board and 1.5 m beyond the board. These two other cameras were oblique to the take-off board. These assistive cameras were employed to aid visual recognition of important events and address any possible occlusions of anatomical features. No coordinate reconstruction was done with the aid of auxiliary camera footage.

All cameras were set manually at the same focus, exposure, shutter speed, and white balance before the start of recording. This was done to reduce the variation between lighting conditions. Then, to ensure geometric consistency of the primary recording plane, the camera alignment and distance relative to the runway were measured using a laser rangefinder and a levelling instrument. All the cameras were placed on the tripod to avoid vibration and the drift of the perspective during data gathering.

Recording was initiated about 5–10 s prior to the entry of any athlete into the capture area and 5–10 s upon leaving the capture area. In order to assure the space accuracy, 2D calibration was administered at the start and end of every session with a calibration frame of 1.20 m x 1.20 m that was positioned inside take-off area and placed parallel to the plane of the primary camera. Wind speed data were not available for all trials, as recordings were conducted under official competition conditions without direct access to wind measurements. Therefore, potential effects of wind on approach velocity cannot be fully excluded.

### 2.4 Kinematic analysis

**Figure 2:**
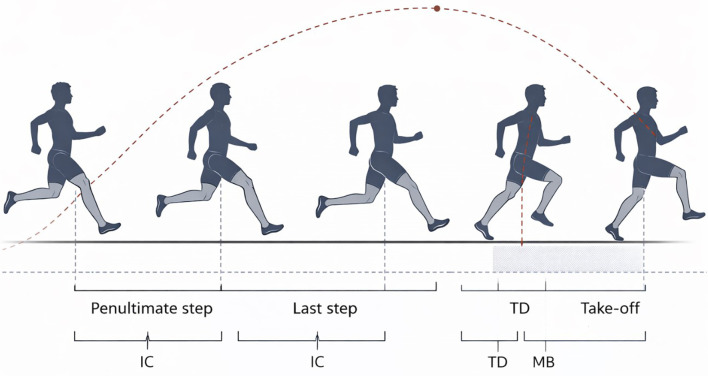
Schematic sagittal-plane representation of the penultimate step, last step, and take-off support phase in the long jump. Key analyzed events are indicated: initial contact (IC) of the penultimate and last steps, touchdown on the board (TD), maximum braking (MB; frame of minimum knee angle), and take-off (TO). The dashed curve illustrates the schematic center-of-mass (COM) trajectory.

Video recordings were brought into Dartfish (version 10.0) and Kinovea. This was performed to enable inspection and the identification of major temporal events during the take-off phase including penultimate foot contact, last foot contact, touchdown (TD) with the take-off board, maximum braking (MB), and take-off (TO) (**Figure 2**). All definitions of events were made on recognizable kinematic criteria of the sagittal plane.

Afterwards, digitization and coordinate extraction were performed using Peak Motus (version 9.0). Anatomical landmarks defining the trunk and lower-limb segments (hip, knee, ankle, toe) were manually digitized frame-by-frame relative to the calibrated image plane. Whole-body center-of-mass (COM) position was estimated in the sagittal plane using a linked-segment model based on standard anthropometric parameters derived from established datasets (Dempster, 1955; De Leva, 1996). Horizontal and vertical COM displacement and velocity components were then derived from the filtered coordinate data.

To minimize random error associated with manual digitization, all trials were analyzed by the same experienced operator. Each trial was digitized on two separate occasions separated by at least 7 days, and the mean of the two digitization sets was used for statistical analysis. To assess the reliability of the digitization procedure, repeated digitization of randomly selected trials was performed. The intra-class correlation coefficient (ICC) for key kinematic variables ranged from 0.92 to 0.96, indicating high reliability of the manual digitization process. The digitization protocol followed standard procedures commonly adopted in applied two-dimensional sports biomechanics analyses.

COM coordinate time-series data were filtered using a fourth-order zero-lag Butterworth low-pass filter with a cut-off frequency of 6 Hz. The cut-off frequency was selected based on established practice in sagittal-plane analyses of explosive lower-limb movements and confirmed through residual analysis to balance signal smoothing and preservation of movement dynamics. Filtered coordinates were used to compute horizontal (Vx), vertical (Vy), and resultant (Σ) velocity variables.

### 2.5 Key technical variables

Technical variables were categorized into three groups: (1) late-approach spatiotemporal parameters, (2) take-off velocity and conversion variables, and (3) sagittal-plane joint kinematics of the take-off leg. Operational definitions are provided in **Table 2**. In addition, temporal characteristics of the take-off support phase were calculated, including braking phase time, propulsion phase time, total contact time, velocity loss rate, and change in COM height. These variables were included to provide additional insight into stretch–shortening cycle behavior during the take-off phase.

**Table 2:** Classification and operational definitions of the kinematic variables analyzed.

Category	Variable (unit)	Operational definition
Late-approach spatiotemporal	Penultimate step length (m)	Horizontal distance between initial contact of the penultimate step and initial contact of the last step.
	Penultimate step time (s)	Time interval between initial contact of the penultimate step and initial contact of the last step.
	Penultimate step velocity (m·s^-^¹)	Mean horizontal step velocity calculated as step length ÷ step time.
	Last step length (m)	Horizontal distance between initial contact of the last step and touchdown (TD) of the take-off foot on the board.
	Last step time (s)	Time interval between initial contact of the last step and touchdown (TD).
	Last step velocity (m·s^-^¹)	Mean horizontal step velocity calculated as step length ÷ step time.
Take-off velocity and conversion variables	Horizontal velocity at touchdown, Vx_TD (m·s^-^¹)	Horizontal center-of-mass (COM) velocity at touchdown (TD).
	Vertical velocity at touchdown, Vy_TD (m·s^-^¹)	Vertical COM velocity at touchdown (TD).
	Resultant velocity at touchdown, ΣTD (m·s^-^¹)	Resultant COM velocity at touchdown: √(Vx_TD² + Vy_TD²).
	Horizontal velocity at take-off, Vx_TO (m·s^-^¹)	Horizontal COM velocity at take-off (TO; final frame prior to toe-off).
	Vertical velocity at take-off, Vy_TO (m·s^-^¹)	Vertical COM velocity at take-off (TO).
	Resultant velocity at take-off, ΣTO (m·s^-^¹)	Resultant COM velocity at take-off: √(Vx_TO² + Vy_TO²).
	Velocity utilisation ratio (ΣTO/ΣTD)	Ratio of resultant velocity at take-off to resultant velocity at touchdown, representing velocity retention and conversion efficiency during ground contact.
	Braking phase time (s)	Time interval between touchdown (TD) of the take-off foot on the board and maximum braking (MB), representing the eccentric deceleration phase of ground contact.
	Propulsion phase time (s)	Time interval between maximum braking (MB) and take-off (TO), representing the concentric push-off phase of the take-off support.
	Total contact time (s)	Total duration of ground contact during the take-off step, calculated as the time interval between touchdown (TD) and take-off (TO).
	Velocity loss rate (%)	Percentage reduction in resultant center-of-mass velocity during ground contact, calculated as ((ΣTD − ΣTO)/ΣTD) × 100.
	Change in COM height (m)	Vertical displacement of the center of mass from touchdown (TD) to maximum braking (MB), representing the preparatory lowering of the body prior to propulsion.
Joint kinematics (take-off leg, sagittal plane)	Take-off angle (°)	Angle between the resultant COM velocity vector and the horizontal at take-off, calculated as tan^-^¹ (Vy_TO/Vx_TO).
	Hip angle at TD (°)	Sagittal-plane hip joint angle at touchdown (TD).
	Knee angle at TD (°)	Sagittal-plane knee joint angle at touchdown (TD).
	Ankle angle at TD (°)	Sagittal-plane ankle joint angle at touchdown (TD).
	Hip angle at MB (°)	Sagittal-plane hip joint angle at maximum braking (MB; frame of peak knee flexion during support).
	Knee angle at MB (°)	Sagittal-plane knee joint angle at maximum braking (MB).
	Ankle angle at MB (°)	Sagittal-plane ankle joint angle at maximum braking (MB).
	Hip angle at TO (°)	Sagittal-plane hip joint angle at take-off (TO; final frame prior to toe-off).
	Knee angle at TO (°)	Sagittal-plane knee joint angle at take-off (TO).

TD, touchdown; MB, maximum braking; TO, take-off; COM, center of mass.

### 2.6 Statistical analysis

All kinematic variables derived from motion analysis were organized in Microsoft Excel (Microsoft Corp., Redmond, WA, USA) for preliminary data management, and subsequently analyzed using SPSS version 26.0 (IBM Corp., Armonk, NY, USA). Data are presented as mean ± standard deviation (M ± SD) to facilitate comparison with previous literature in sports biomechanics. When variables did not meet normality assumptions, non-parametric tests were applied, and results should be interpreted accordingly. Normality of all variables was assessed using the Shapiro–Wilk test. Homogeneity of variances was evaluated using Levene’s test. For variables meeting parametric assumptions, independent-samples t-tests were used to examine between-group differences. When assumptions were violated, the Mann–Whitney U test was applied. Statistical significance was set at p < 0.05 (two-tailed). Effect sizes were calculated using Cohen’s d for parametric comparisons. For non-parametric analyses, effect sizes were interpreted with caution, as Cohen’s d is based on group means and standard deviations. Effect sizes were interpreted as trivial (<0.20), small (0.20–0.49), moderate (0.50–0.79), or large (≥0.80). Associations between kinematic variables and jump distance were assessed using Pearson’s correlation coefficient (r) for normally distributed data and Spearman’s rank correlation coefficient (ρ) when normality was not satisfied. Correlation strength was interpreted as low (|r| < 0.30), moderate (0.30 ≤ |r| < 0.50), substantial (0.50 ≤ |r| < 0.80), or high (|r| ≥ 0.80). Given the exploratory nature of the analysis and the relatively small sample size, no formal adjustment for multiple comparisons was applied, as such corrections may increase the risk of Type II error. Instead, emphasis was placed on effect sizes and confidence intervals to aid practical interpretation. However, the potential for inflated Type I error due to multiple comparisons should be considered when interpreting the results. Due to the relatively small sample size and the elite nature of the population, no *a priori* power analysis was conducted. However, a *post hoc* sensitivity analysis indicated that the study was adequately powered to detect large effect sizes (d ≥ 0.80) at α = 0.05. Therefore, non-significant findings, particularly those associated with small-to-moderate effect sizes, should be interpreted with caution.

## 3 Results

### 3.1 Take-off velocity and angular characteristics

**Figure 3:**
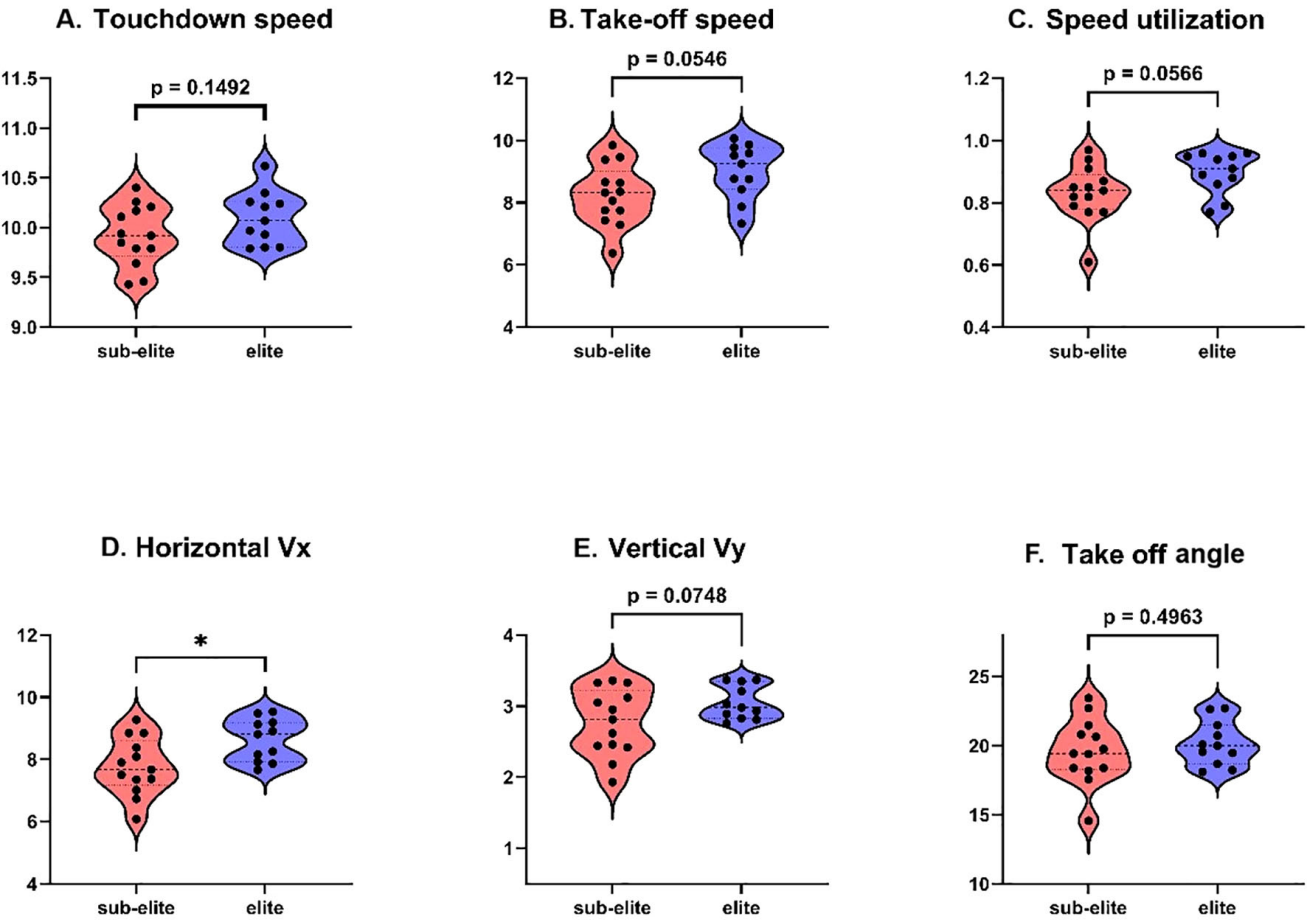
Group comparisons of take-off velocity-related variables and take-off angle in elite and sub-elite male long jumpers. Violin plots show the distribution of individual values for **(A)** resultant velocity at touchdown, **(B)** resultant velocity at take-off, **(C)** velocity utilization ratio, **(D)** horizontal take-off velocity, **(E)** vertical take-off velocity, and **(F)** take-off angle. Black dots represent individual athletes. Horizontal internal lines indicate central tendency and distributional spread. Between-group p-values are displayed above each panel; * indicates a statistically significant difference between groups. Vx, horizontal take-off velocity; Vy, vertical take-off velocity; ΣTD, resultant velocity at touchdown; ΣTO, resultant velocity at take-off.

Descriptive statistics and between-group comparisons are presented in **Table 3** and illustrated in **Figure 3**. The elite group demonstrated a significantly greater horizontal take-off velocity (Vx_TO) compared with the sub-elite group (8.63 ± 0.68 vs. 7.77 ± 0.91 m·s^-^¹; p = 0.018; d = 1.05; 95% CI [0.16, 1.55]). No other velocity variable reached statistical significance. Resultant velocity at take-off (ΣTO) was higher in the elite group (9.02 ± 0.88 vs. 8.26 ± 0.97 m·s^-^¹), with a moderate-to-large effect size (d = 0.82), although the between-group difference did not reach statistical significance (p = 0.055). Similarly, the velocity utilization ratio (ΣTO/ΣTD) showed a moderate effect size (d = 0.80). But this was not statistically significant (p = 0.057). Although these variables did not reach statistical significance, the moderate-to-large effect sizes suggest potentially meaningful performance differences that may not have been detected due to the relatively small sample size. The vertical take-off velocity (Vy_TO) exhibited a moderate effect size (d = 0.73). The higher values were found in the elite group (3.05 ± 0.24 vs. 2.77 ± 0.47 m·s^-^¹), but the difference was not statistically significant (p = 0.075). No between-group difference was observed in take-off angle (20.15 ± 1.60° vs. 19.59 ± 2.33°; p = 0.496; d = 0.28).

**Table 3:** Between-group comparison of take-off velocity components and angular characteristics in elite and sub-elite male long jumpers.

Variable	Elite (n = 11)	Sub-elite (n = 13)	t	p (two-tailed)	Cohen’s d	95% CI of mean difference (elite − sub-elite)
Resultant velocity at touchdown ∑TD (m·s^-^¹)	10.09 ± 0.27	9.92 ± 0.30	1.476	0.149	0.61	[−0.07, 0.42]
Resultant velocity at take-off ∑TO (m·s^-^¹)	9.02 ± 0.88	8.26 ± 0.97	2.011	0.055	0.82	[−0.02, 1.55]
Velocity utilization ratio (∑TO/∑TD)	0.8965 ± 0.0683	0.8320 ± 0.0891	1.962	0.057	0.8	[−0.0037, 0.1328]
Horizontal velocity at take-off Vx (m·s^-^¹)	8.63 ± 0.68	7.77 ± 0.91	2.554	0.018	1.05	[0.16, 1.55]
Vertical velocity at take-off Vy (m·s^-^¹)	3.05 ± 0.24	2.77 ± 0.47	1.884	0.075	0.73	[−0.03, 0.59]
Take-off angle (°)	20.15 ± 1.60	19.59 ± 2.33	0.671	0.496	0.28	[−1.17, 2.29]

Values are presented as mean ± SD. Independent-samples t-tests were used unless otherwise indicated. Cohen’s d represents effect size. CI, confidence interval. Welch’s correction was applied in cases where the assumption of homogeneity of variance was violated.

### 3.2 Ground contact phase characteristics

To further examine stretch–shortening cycle behavior during take-off, ground contact phase characteristics were analyzed, including braking phase time, propulsion phase time, total contact time, velocity loss rate, and change in center-of-mass height (**Table 4**). These variables provide insight into the efficiency of eccentric–concentric force transition during the take-off support phase. Elite athletes demonstrated slightly shorter braking phases and lower velocity loss rates than sub-elite athletes, suggesting more effective preservation of horizontal momentum during ground contact. Although the difference in velocity loss rate approached statistical significance (p = 0.057), total contact time was comparable between groups, indicating similar temporal constraints of the stretch–shortening cycle during the take-off phase.

**Table 4:** Ground contact phase characteristics during take-off in elite and sub-elite male long jumpers.

Variable	Elite (n = 11)	Sub-elite (n = 13)	t	p (two-tailed)
Braking phase time (s)	0.054 ± 0.009	0.056 ± 0.009	−0.68	0.502
Propulsion phase time (s)	0.056 ± 0.008	0.060 ± 0.010	−1.01	0.323
Total contact time (s)	0.110 ± 0.012	0.116 ± 0.013	−1.32	0.201
Velocity loss rate (%)	10.4 ± 2.3	12.1 ± 2.7	−2.01	0.057
Change in COM height (m)	0.192 ± 0.034	0.206 ± 0.039	−0.96	0.349

Values are presented as mean ± SD. Independent-samples t-tests were used to compare groups. COM = center of mass.

### 3.3 Late-approach spatiotemporal characteristics

The between-group comparisons for penultimate and last strides are summarized in **Table 5**. There was no statistically significant difference which was observed in step length, step time, or step velocity for the penultimate and last stride. Effect sizes were insignificant for most variables (|d| ≤ 0.19), with the exception of penultimate-step velocity. Penultimate-step velocity showed a small effect size (d = 0.19) and was not statistically significant (p = 0.648). All 95% confidence intervals for the mean differences included zero. It indicated substantial overlap between groups across late-approach spatiotemporal variables.

**Table 5:** Between-group comparison of penultimate and last stride spatiotemporal variables in elite and sub-elite male long jumpers.

Variable	Elite (n = 11)	Sub-elite (n = 13)	t	p (two-tailed)	Cohen’s d	95% CI of mean difference (elite − sub-elite)
Penultimate step length (m)	2.31 ± 0.08	2.33 ± 0.12	−0.588	0.563	0.11	[−0.12, 0.07]
Penultimate step time (s)	0.21 ± 0.02	0.21 ± 0.02	−0.588	0.563	0.11	[−0.016, 0.008]
Penultimate step velocity (m·s^-^¹)	11.19 ± 0.50	11.08 ± 0.64	0.463	0.648	0.19	[−0.617, 0.992]
Last step length (m)	2.13 ± 0.12	2.15 ± 0.15	−0.316	0.755	−0.13	[−0.14, 0.10]
Last step time (s)	0.19 ± 0.02	0.19 ± 0.02	0.095	0.925	0.04	[−0.013, 0.014]
Last step velocity (m·s^-^¹)	11.44 ± 0.72	11.54 ± 0.38	−0.452	0.656	−0.19	[−0.60, 0.39]

Values are presented as mean ± SD. Cohen’s d represents effect size. CI, confidence interval.

### 3.4 Sagittal-plane joint angles during take-off

**Figure 4:**
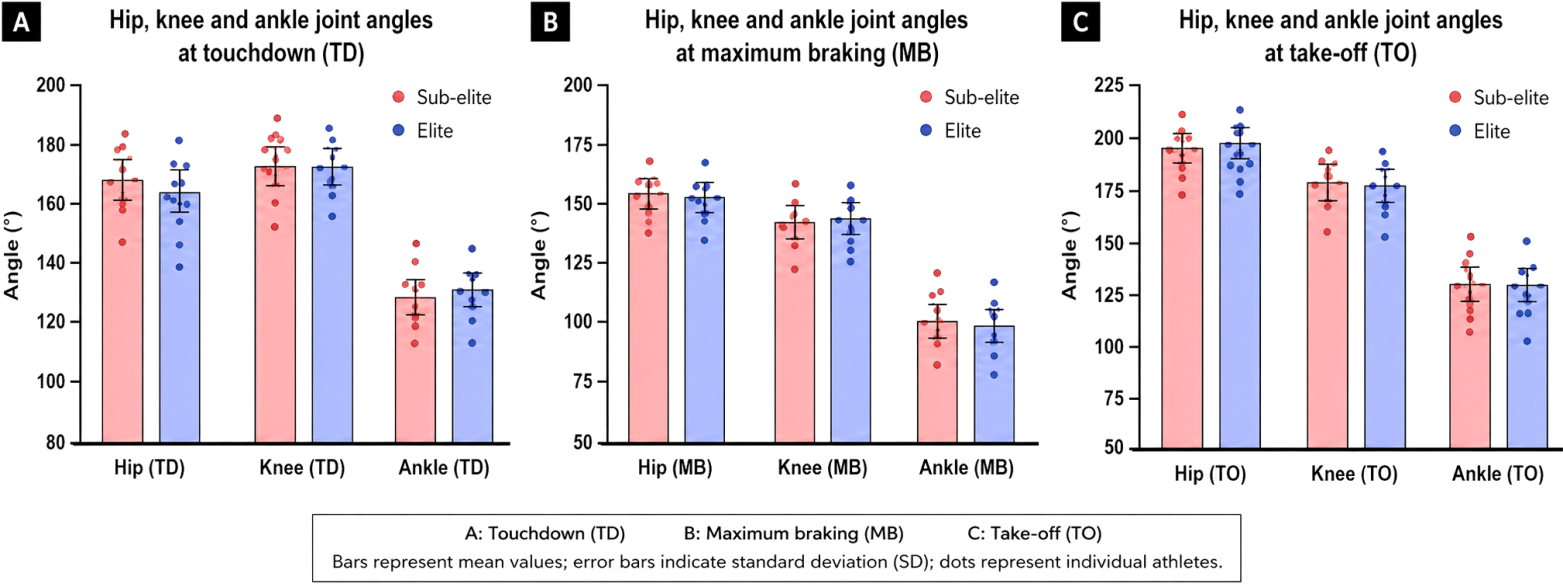
Sagittal-plane hip, knee, and ankle joint angles at **(A)** touchdown (TD), **(B)** maximum braking (MB), and **(C)** take-off (TO) in elite and sub-elite male long jumpers. Bars represent mean values; error bars indicate standard deviation (SD); dots represent individual athletes.

Joint-angle comparisons at touchdown (TD), maximum braking (MB), and take-off (TO) are presented in **Table 6** and illustrated in **Figure 4**. No statistically significant between-group differences were detected for hip, knee, or ankle joint angles at any analyzed event (all p > 0.05). Effect sizes were trivial to small for most comparisons, with moderate magnitudes observed for hip angle at touchdown (d = −0.40) and ankle angle at take-off (d = −0.44), although corresponding confidence intervals spanned zero. Overall, sagittal-plane joint-angle magnitudes were comparable between elite and sub-elite athletes across TD, MB, and TO.

**Table 6:** Between-group comparison of sagittal-plane hip, knee, and ankle joint angles at touchdown (TD), maximum braking (MB), and take-off (TO) in elite and sub-elite male long jumpers.

Angle (°)	Phase	Elite (M ± SD)	Sub-elite (M ± SD)	t	p (two-tailed)	Cohen’s d	95% CI (mean difference, elite − sub-elite)
Hip joint	Touchdown	161.08 ± 10.53	164.21 ± 4.77	−0.963	0.346	−0.40	[−9.86, 3.60]
	Maximum braking	154.57 ± 6.50	154.41 ± 8.11	0.054	0.957	−0.38	[−6.14, 6.47]
	Take-off	195.35 ± 8.51	194.44 ± 6.86	0.289	0.775	0.12	[−5.60, 7.41]
Knee joint	Touchdown	169.72 ± 3.43	169.60 ± 4.05	0.076	0.94	0.03	[−3.09, 3.33]
	Maximum braking	141.98 ± 4.59	140.42 ± 6.39	0.654	0.52	0.28	[−3.41, 6.54]
	Take-off	172.82 ± 6.38	173.83 ± 4.76	−0.445	0.661	−0.18	[−5.73, 3.71]
Ankle joint	Touchdown	128.23 ± 2.98	128.12 ± 6.87	0.053	0.958	0.02	[−4.33, 4.56]
	Maximum braking	101.05 ± 6.75	102.08 ± 6.39	−0.384	0.705	−0.16	[−6.60, 4.54]
	Take-off	130.75 ± 7.64	133.95 ± 6.89	−1.072	0.296	−0.44	[−9.44, 3.03]

### 3.5 Correlation analysis between take-off parameters and jump distance

**Figure 5:**
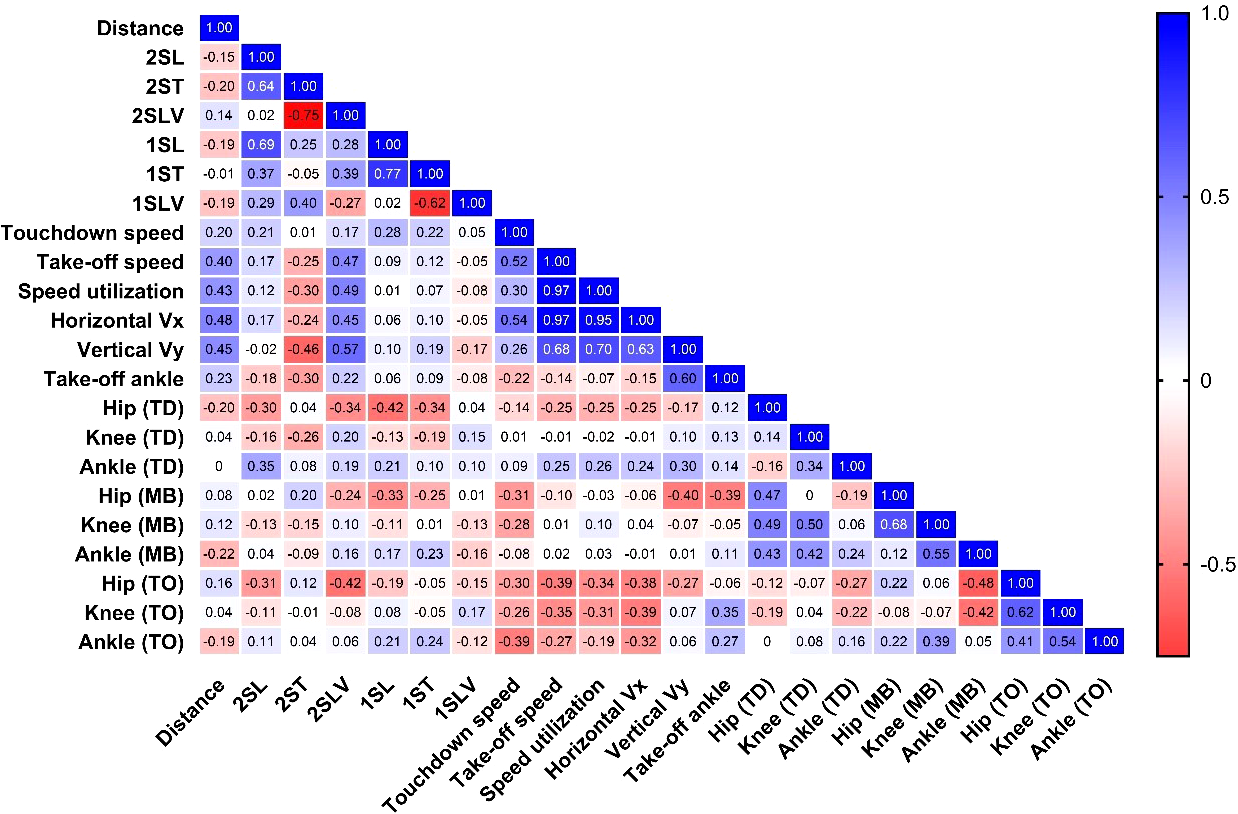
Correlation matrix illustrating relationships between jump distance and selected kinematic variables in male long jumpers. Values represent Pearson’s correlation coefficients (r). Color scale indicates strength and direction of association (blue = positive; red = negative). 2SL, penultimate step length; 2ST, penultimate step time; 2SLV, penultimate step velocity; 1SL, last step length; 1ST, last step time; 1SLV, last step velocity; Vx, horizontal take-off velocity; Vy, vertical take-off velocity; ΣTO, resultant take-off velocity; ΣTO/ΣTD, velocity utilization ratio; TD, touchdown; MB, maximum braking; TO, take-off.

Correlation coefficients are presented in **Figure 5**. Jump distance showed moderate positive relationships with the horizontal take-off velocity (r = 0.48), vertical take-off velocity (r = 0.45), resultant take-off velocity (r = 0.40) and velocity utilization ratio (r = 0.43). The correlation between the take-off angle and the distance of the jump was not high (r = 0.23). Strong interrelationships were found between velocity-related variables. Velocity utilization (r = 0.97) and horizontal take-off velocity (r = 0.97) had a strong correlation with resultant take-off velocity. The use of velocity was also highly related to horizontal take-off velocity (r = 0.95). Vertical take-off velocity was found to have moderate relationships with all the velocity variables (r = 0.63-0.70). Because resultant take-off velocity is mathematically derived from horizontal and vertical velocity components, Strong interrelationships among velocity variables are expected, as resultant take-off velocity is mathematically derived from its horizontal and vertical components. Therefore, these associations should be interpreted with caution, as they partly reflect inherent mathematical dependency rather than independent physiological mechanisms.

Late-approach spatiotemporal variables demonstrated weaker direct associations with jump distance but showed moderate relationships with take-off velocity measures. For example, penultimate-step velocity was moderately correlated with vertical take-off velocity (r = 0.57) and velocity utilization (r = 0.49). Step time and step velocity were inversely correlated for both strides (e.g., penultimate: r = −0.75; last: r = −0.62). Associations between joint angles and jump distance were generally small. The ankle angle at take-off was moderately correlated with vertical take-off velocity (r = 0.60).

**Figure 6:**
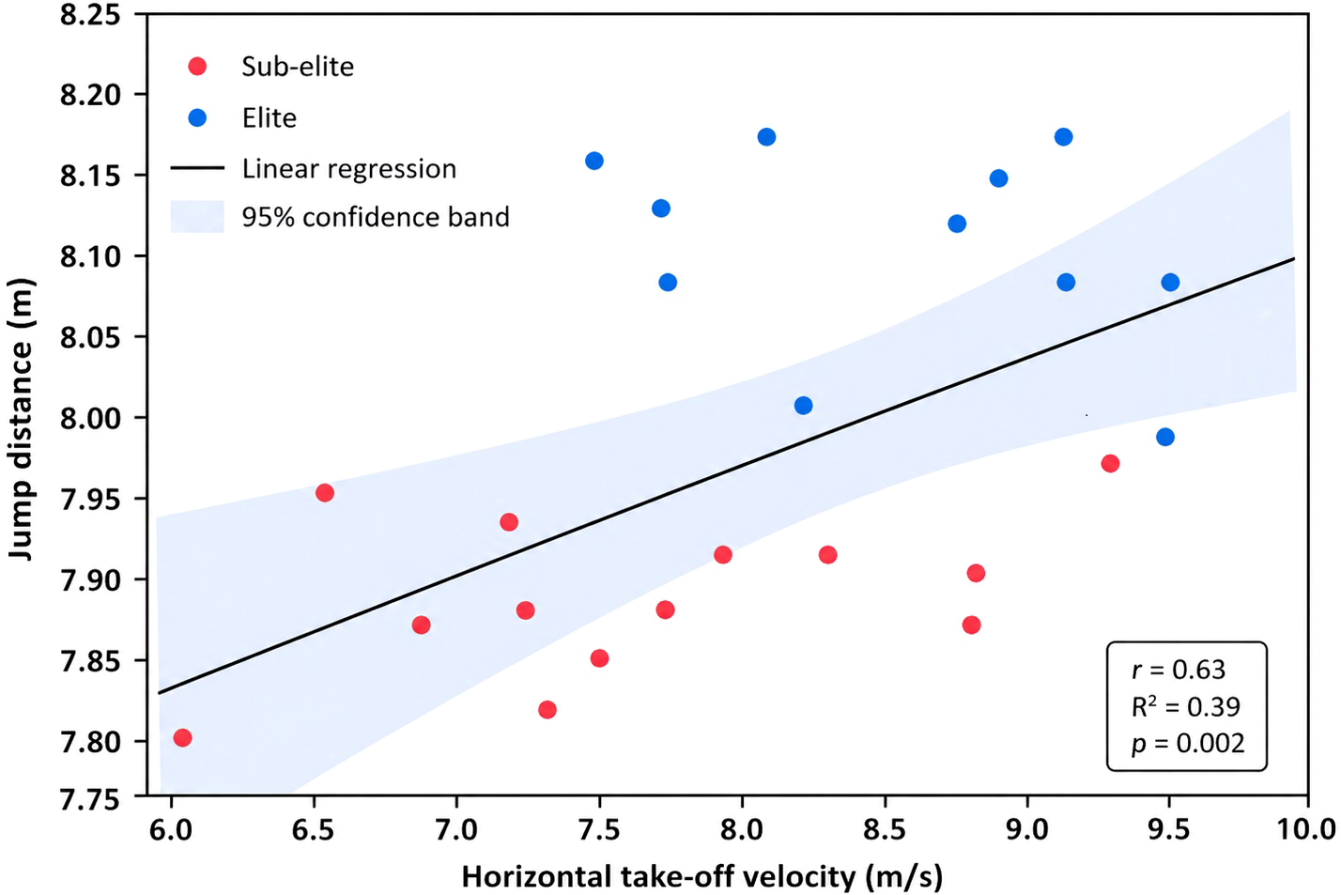
Relationship between horizontal take-off velocity (Vx) and jump distance in elite and sub-elite male long jumpers. Each point represents an individual athlete. The solid line represents the linear regression, with shaded area indicating the 95% confidence interval.

A scatter plot illustrating the relationship between horizontal take-off velocity and jump distance is presented in **Figure 6**. The distribution of points demonstrates a positive association between these variables, indicating that athletes with greater horizontal velocity at take-off tend to achieve longer jump distances. This visual pattern is consistent with the moderate correlation observed between horizontal take-off velocity and performance (r = 0.48).

## 4 Discussion

The current competition based research showed that there was a primary difference in horizontal take-off velocity between groups, while other kinematic variables showed largely similar patterns in elite and sub-elite long jumpers. The elite jumpers in our study had a much higher horizontal velocity at take-off. Consistent with the current results, the male jumpers who won the medal had higher final-approach speed and higher horizontal take-off velocity than the sub-elite ones. These gains were not accompanied by variation in the take-off angle or contact time. Thus, key postural markers at take-off were essentially the same for both groups (Turner et al., 2025). It resonates with the opinion that maximizing speed instead of angle change is the major tactic in the long jump. The ratio of the velocity utilization appears to be greater among the elites and is associated with their high capabilities to maintain the momentum (Zhang et al., 2025). In practice, this meant that although both groups lost some horizontal speed as they generated vertical impulse, the elite jumpers minimized this loss. All these findings tend to indicate that elite sportsmen were more concerned with faster speeds and more efficient maintenance of speed, and not radical technique shifts at take-off. It aligns with models that have made horizontal velocity the most important determinant of jump distance. It is important to acknowledge that horizontal take-off velocity is inherently related to jump distance due to the underlying mechanics of projectile motion. Therefore, the observed between-group difference in horizontal velocity should not be interpreted as an entirely independent determinant of performance. Rather, the present findings highlight differences in how effectively athletes preserve and utilize approach velocity during the take-off phase under competitive conditions. These findings are consistent with classical long jump biomechanics literature, which emphasizes the importance of approach velocity and its preservation during take-off (Hay, 1993; Linthorne, 2008). Although the importance of horizontal velocity in long jump performance is well established, the present findings provide additional insight into how this velocity is preserved during the take-off phase among closely matched athletes. Notably, the similarity in stride parameters and joint kinematics between groups suggests that performance differences may be driven more by velocity maintenance and efficiency of force application rather than distinct technical differences in movement patterns.

From an exercise physiology perspective, these findings highlight the importance of neuromuscular coordination and stretch–shortening cycle (SSC) efficiency during explosive athletic performance. The take-off phase requires rapid eccentric–concentric muscle interaction within the lower-limb muscle–tendon units, allowing athletes to absorb mechanical energy during braking and rapidly reapply force during push-off. Elite jumpers’ ability to maintain greater horizontal velocity at take-off may therefore be associated with greater neuromuscular activation, higher rate of force development, and more effective utilization of elastic energy, although these variables were not directly assessed. These physiological characteristics are central to explosive performance and are widely recognized as key determinants of success in high-intensity stretch–shortening cycle activities.

It is important to note that there were only slight differences in vertical take-off velocity between elite and sub-elite jumpers. The vertical COM velocity (of male elites) showed no significant difference in our data, but the effect was smaller than that for horizontal velocity. This trend is consistent with past biomechanical studies that suggest elite male long jumpers gain the majority of their performance benefit through an increase in speed of approach and take-off and not the take-off angle (Mangi et al., 2025). Some of the reports indicate that in females, elites might use slightly higher vertical impulse than men but our sample, which consisted mostly of males, indicated that there was no significant difference in the take-off angles of the two groups (Pavlovic, 2016). It implied that both groups were already using near-ideal trajectories. The differences in length of jump was driven largely by differences in speed.

Stride kinematics established that benefits of elite jumpers were due to speed, and not changes of stride length. Our study observed no significant differences in last-stride lengths between groups. But the elites always ran the last strides at a faster rate. These results are in agreement with the competition analysis. The medalists have an increased speed in their final stride (Padullés et al., 2019). The data imply that the elite athletes had a greater top-end speed and a superior ability to resist deceleration over multiple steps. By contrast, sub-elites exhibited similar stride lengths but slightly lower speeds. It suggested that their technical focus may need to target speed conservation rather than altering step pattern. Practically, the horizontal velocity at the last foot-strike was approximately 0.3-0.4 m/s greater on elites than on others. This was determined to be in line with the fact that elite jumpers identify the board in an effective manner and do not decelerate because of last minute changes.

Joint-angle analysis indicated broadly similar take-off postures across groups. Both elite and sub-elite jumpers exhibited the typical long jump kinematic pattern. At touchdown the knee was nearly straight (peak flexion angle of 167–170°) and then knee and hip extended rapidly to generate lift. Our knee-angle data align with past reports of elite jumpers (~167° at touchdown), and we found no systematic group differences in peak knee flexion or trunk lean during stance (Bridgett and Linthorne, 2006). The take-off contact time (~0.11–0.12 s) was also virtually identical for both groups. It indicated that they utilized the stretch–shortening cycle (SSC) over comparable time intervals. Collectively, these similarities imply that both groups adopted an efficient biomechanical posture.

Our findings align closely with contemporary biomechanics research on the long jump. A recent systematic review reported that elite male long jumpers exhibit both higher approach speeds and higher take-off speeds than less successful competitors (Turner et al., 2025). Specifically, it found that medallists had ~0.29 m/s greater horizontal take-off velocity and greater approach-section speeds, with no significant change in take-off angle, matching our results. Similarly, Jin et al. showed that elite jumpers benefit from improved swing-leg braking, which enhances support-leg extension velocity. Additionally, recent neuromuscular investigations emphasize the role of knee extensors and gluteals in generating vertical impulse (Jin et al., 2024). For example, Yang et al. used musculoskeletal modeling to show that vastus, soleus and gluteus maximus account for approximately 73% of the vertical velocity gain at take-off. Likewise, the quadriceps also mediate most of the horizontal braking. This highlights that the ability of these muscles to engage quickly under load likely underlies the velocity differences we observed (Yang et al., 2023).

In terms of SSC and force production, our results echo the broader consensus that reactive strength and stiffness are critical for jump performance. The elite jumpers’ superior speed conservation suggests a more effective eccentric–concentric muscle–tendon interaction during the penultimate and take-off steps. This is supported by recent training studies: for instance, Xu et al. demonstrated that long jumpers who undertook combined strength and plyometric training increased their leg stiffness and elastic energy use. It facilitated more efficient force transmission during the penultimate and take-off steps (Xu et al., 2025b). It, thereby, aids in the maintenance of horizontal velocity and enhancement of take-off efficiency. In short, jumping faster into take-off demands very rapid force generation to decelerate and reaccelerate the body. Our findings are congruent with the idea that RFD and stiffness improvements (rather than pure strength gains) are most important for explosive jumps (Dodd and Alvar, 2007; Mihalik et al., 2008). Elite jumpers’ ability to maintain velocity likely reflects superior neuromuscular qualities – greater RFD, tendon stiffness, and neuromuscular coordination – all underpinned by an effective SSC. In addition, explosive strength likely contributes to take-off performance by enabling rapid force production in both horizontal and vertical directions during the brief ground contact phase.

The stretch–shortening cycle (SSC) is central to understanding take-off mechanics. The last ground contact includes rapid eccentric loading of muscles and then a concentric push-off, which stores and releases elastic energy (Pedley et al., 2022). Elite jumpers, in our competition, seem to use the SSC better and maintain momentum as they develop the required vertical force. This may reflect an increased musculotendinous stiffness and reactive strength of elite athletes. Recent evidence underscores that jumpers with training-induced increases in SSC efficiency can better maintain approach velocity at take-off (Xu et al., 2025a). Moreover, neural factors such as pre-activation and reflex potentiation may contribute; although we did not measure EMG, it is plausible that elite athletes have superior neuromuscular timing. The net effect under competition conditions is that the take-off leg’s muscle preloads and subsequent recoil are finely tuned in elites and yields higher net velocity. This aligns with the broader observation of our study that medal-winning jumpers across events consistently demonstrate better velocity maintenance and higher explosive force profiles. Practically, our results complement the fact that the take-off stage of elite jumpers is defined by higher raw velocity, as well as better use of SSC, which in combination increases the distance of the jump. However, these physiological interpretations should be considered indirect, as variables such as rate of force development, tendon stiffness, and neuromuscular activation were not directly measured in the present study.

Several limitations should be acknowledged. First, the use of two-dimensional video analysis may not fully capture three-dimensional joint mechanics during the take-off phase. Second, the relatively small sample size reflects the limited availability of athletes performing within a narrow elite performance range. While this enhances group homogeneity, it reduces statistical power for detecting smaller between-group differences. Furthermore, the analysis involved multiple statistical comparisons across a large number of variables, which increases the risk of Type I error. Although effect sizes and confidence intervals were reported to support interpretation, the findings should be interpreted with appropriate caution. Thirdly, kinetic variables such as ground reaction forces and muscle activation patterns were not measured and should be considered in future investigations. In addition, data were collected across multiple competitions held over two seasons, and environmental factors such as wind, temperature, and runway conditions were not controlled. These factors may have influenced performance variability and should be considered when interpreting the results. Lastly, the use of two-dimensional (2D) sagittal-plane analysis limits the ability to capture three-dimensional joint mechanics, including transverse and frontal plane movements, foot placement variability, trunk rotation, and arm-swing contributions. These factors are known to influence take-off performance and may contribute to variability in force generation and body positioning. As a result, the present analysis may underestimate the contribution of multi-planar movements to performance differences, particularly in aspects related to coordination and angular momentum. Therefore, the findings should be interpreted primarily within the context of sagittal-plane kinematics.

## 5 Conclusions

This competition-based analysis demonstrated that elite male long jumpers differentiate themselves from sub-elite athletes primarily through greater horizontal take-off velocity and more efficient preservation of approach speed. Late-approach stride characteristics and sagittal-plane joint kinematics were largely similar between groups. These findings emphasize the importance of maximizing approach velocity and developing neuromuscular qualities that support efficient stretch–shortening cycle function during the take-off phase.

## Data Availability

The raw data supporting the conclusions of this article will be made available by the authors, without undue reservation.
